# Planning, Implementing, and Running a Multicentre Preterm Birth Study with Biobank Resources in Brazil: The Preterm SAMBA Study

**DOI:** 10.1155/2019/5476350

**Published:** 2019-01-16

**Authors:** Renato T. Souza, Jose G. Cecatti, Maria L. Costa, Jussara Mayrink, Rodolfo C. Pacagnella, Renato Passini, Kleber G. Franchini, Francisco E. Feitosa, Iracema M. Calderon, Edilberto A. Rocha Filho, Débora F. Leite, Janete Vettorazzi, Louise C. Kenny, Philip N. Baker, The Preterm SAMBA Study Group

**Affiliations:** ^1^Department of Obstetrics and Gynecology, University of Campinas (UNICAMP), School of Medical Sciences, Campinas, SP, Brazil; ^2^LNBio-Brazilian National Laboratory of Biosciences and University of Campinas (UNICAMP) School of Medical Sciences, University of Campinas (UNICAMP), Campinas, SP, Brazil; ^3^MEAC–Maternity School of The Federal University of Ceará, Fortaleza, CE, Brazil; ^4^Department of Obstetrics and Gynecology, Botucatu Medical School, Sao Paulo State University–Unesp, Botucatu, Brazil; ^5^Department of Maternal and Child Health, Maternity of The Clinics Hospital, Federal University of Pernambuco, Recife, PE, Brazil; ^6^Department of Obstetrics and Gynecology, Maternity of The Clinics Hospital, Federal University of RS, Porto Alegre, RS, Brazil; ^7^Irish Centre for Fetal and Neonatal Translational Research (INFANT), Department of Obstetrics and Gynaecology, University College Cork, Cork, Ireland; ^8^College of Medicine, Biological Sciences and Psychology, University of Leicester, Leicester, UK; ^9^Gravida: National Centre for Growth & Development, Auckland, New Zealand

## Abstract

**Background:**

Our aim was to describe the steps in planning, implementing, and running a multicentre cohort study of maternal and perinatal health using a high-quality biobank comprised of maternal serum, plasma, and hair samples collected from five sites in Brazil. The Preterm SAMBA study, conducted by the Brazilian Network for Studies on Reproductive and Perinatal Health, was an innovative approach used to identify women at higher risk for preterm birth. It is also of great importance in the study of other maternal and perinatal complications in the context of Brazil, which is a middle-income country.

**Methods:**

We described phases of planning, implementing, and running the Preterm SAMBA study, a multicentre Brazilian cohort study of low-risk nulliparous pregnant women, to validate a set of metabolite biomarkers for preterm birth identified in an external cohort. Procedures and strategies used to plan, implement, and maintain this multicentre preterm birth study are described in detail. Barriers and experience cited in the current narrative are not usually discussed in the scientific literature or published study protocols.

**Results:**

Several barriers and strategies were identified in different phases of the Preterm SAMBA study at different levels of the study framework (steering committee; coordinating and local centres). Strategies implemented and resources used in the study are a legacy of the Brazilian Network, aimed at training collaborators in such complex settings.

**Conclusion:**

The Brazilian Network for Studies on Reproductive and Perinatal Health has gained some experience in conducting a multicentre cohort study using a resourceful biobank which may be helpful to other research groups and maternal/perinatal health networks that plan on employing a similar approach to a similar background.

## 1. Background

The Brazilian Network for Studies on Reproductive and Perinatal Health (BNSRPH) is a national research initiative that has enabled a number of multicentre studies within the country. It has had a major impact on maternal and perinatal health in the last decade [1–4]. The BNSRPH was officially launched in 2008, when twenty-seven institutions from five regions of Brazil agreed to participate in the first multicentre study coordinated by this network. These institutions were provided with funding to perform surveillance of severe maternal morbidity [5–7]. For the conception of this Brazilian initiative [5], it was particularly important to have experience as partners and data collectors in multicentre studies headed by international initiatives such as the University of Cincinnati, USA [8], and most importantly the University of Toronto, Canada [9].

Experience and effectiveness of epidemiological studies have evolved to the possibility of scaling up and planning studies that meet translational research requirements. It is an approach to propose interventions that may have a potential impact on maternal and perinatal health. Current infrastructure has enabled adequate clinical data collection, along with biological sample storage. The Preterm SAMBA project represents this initiative.

The Preterm SAMBA cohort study (Preterm Screening and Metabolomics in Brazil and Auckland) enrolled around 1,150 low-risk nulliparous pregnant women from 5 different Brazilian centres. Participants received follow-up care at three study visits during pregnancy (20, 27, and 37^th^ weeks gestation). Blood and hair samples were collected at 20 weeks of gestational age (+/- 1wk). Comprehensive clinical data including blood pressure levels, BMI (body mass index), medical history, clinical complications such as infectious diseases, ultrasound data, and maternal and perinatal outcomes were standardised, collected, and recorded online in an electronic database. Biological samples were collected, processed, and immediately stored in the laboratory, under specific conditions and following a very precise operational biobank protocol [4]. The main objective was to validate biological predictors of preterm birth based on the metabolomics approach.

Planning, implementing, and running a multicentre preterm birth study with biobank resources in Brazil represents a challenge that should be acknowledged. Experience that is not always reported in research protocols can serve as an example, especially in under-resourced settings where difficulties in pursuing these studies are known to be enormous. We aim to tackle all issues involved in this initiative.

## 2. Methods

### 2.1. Planning

Research projects can only thrive with proper funding. In 2013, the Preterm SAMBA project proposal was submitted to a joint research call from the Bill and Melinda Gates Foundation (BMGF), the Brazilian Ministry of Health and the Brazilian National Research Council (CNPq), an agency under the Ministry of Science and Technology. This grant was part of a program called “Reducing the burden of preterm birth,, an agenda of shared priorities to address the occurrence and consequences of preterm birth through innovative approaches in locations where the burden of disease is heaviest. At that time, it seemed very appropriate to form a partnership with international collaboration. The chosen international group had pioneered the application of metabolomics technologies in maternal and perinatal health [10–13] and could help build a very high-quality biobank.

Interestingly, the full proposal of this study was only developed during the selection process of this research call. Initially, a 5-page letter of intention was presented and selected in the first round. Then, a full proposal was presented for the second round, when a technical examining committee was in charge of assessing proposals and making recommendations. Finally, in the third round the same committee was responsible for checking changes in the proposal, according to previous suggestions and selecting the final grantees. This process allowed for some improvements in the research proposal while the selection process was running.

The Preterm SAMBA project aims to develop a predictive algorithm based on biomarker screening tests (involving both phases of discovery and validation) that may translate into clinical care. Ultimately, a panel of biomarkers can be identified as an early pregnancy screening test to ascertain which pregnant women are at higher risk of developing preterm birth (PTB), thus facilitating timely and appropriate interventions.

### 2.2. Ethics, Consent and Permission

The establishment of systematic sample collection and biobank storage requires compliance with rigorous ethical regulations. Ethical aspects for biorepository/biobank (Resolution № 441/11), sample transport (Resolution № 1306/14), shipping samples abroad to international partners (Resolution № 292/99), and subsequent sample use in future studies/analyses (Resolutions № 347/05 and 441/11) were all regulations considered and respected.

The first step was to submit the research project to the Institutional Review Board (IRB) of the coordinating centre for assessment and approval, followed by its registration in the Brazilian National Research Registry platform (*Plataforma Brasil*), where the project was appreciated by each local IRB of the remaining participating centres. Ethical approval was then confirmed by the National Committee for Ethics in Research (CONEP) through Letter of approval number 912.714 from the IRB of the University of Campinas issued on 12/13/2014. Although the coordinating centre had only most recently approved its Institutional Biobank by the Brazilian National Committee for Ethics in Research, the project started after a biorepository and not a biobank was launched and established, according to Brazilian regulation. However, it benefited from Biobank infrastructure, as well as its technical and human resources, assuring quality control for all necessary procedures. No other participating institution where samples were collected and temporarily stored had obtained formal biobank approval. In this case, the local PI and not the institution was the curator responsible for the samples and periodical renovation of ethical approval as required. A clear and elucidative consent form was applied to all participating women, clarifying that, in addition to trivial points, part of the samples would be shipped abroad and future investigations could be conducted using their samples. It was accepted that these participants could agree partially or fully. Furthermore, another important aspect of ethical consideration was the commitment of international partners to follow Brazilian rules.

**Figure 1:**
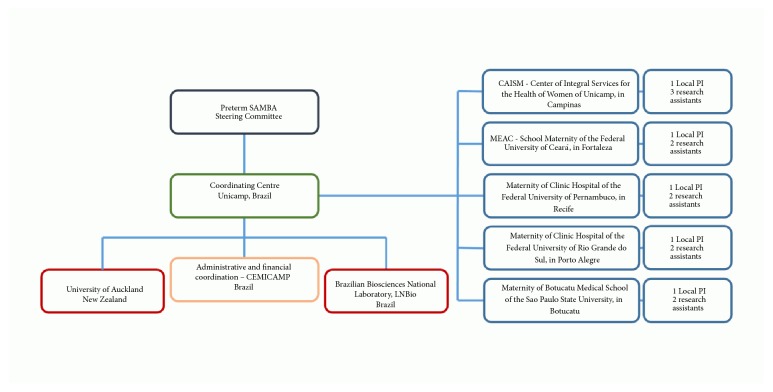
Framework of Preterm SAMBA study.

The coordinating centre (University of Campinas, UNICAMP) and the steering committee had a major role in the framework of the study (Figure 1). The steering committee, represented by the senior investigators of both research groups, played a significant and strategic role throughout the project. The committee was responsible for reviewing, approving and coordinating study data and sample analyses and supervising study progress. As outlined by the flowchart, international partnership was initially established with the University of Auckland, which was hosting the international PI of the study, Prof. P Baker. Shortly afterwards, our second international partner was the University of Cork, Ireland, followed by the University of Leicester, UK. The international PI had moved to the last location during the study.

The five participating centres were selected according to previous performance and commitment to the Brazilian network of multicentre studies, minimal existing infrastructure for biological sample collection and storage, and strategic geographical location, to address populations of different cultural and ethnic background. Each participating centre worked with a local PI and one or more assistant researchers to cover study requirements for subject enrolment, clinical follow-up and laboratory procedures. Team selection is particularly relevant to the development and sustainability of the study. The commitment of the staff exceeded that foreseen by the financial support available and was mainly driven by research interests.

## 3. Results

The general study manager, based in the coordinating centre in Brazil, visited the University of Auckland and University of Cork before implementing the study. Experience and know-how were shared following standard protocols used in the SCOPE and IMPROvED studies [14, 15] by the Preterm SAMBA international partners. It was crucial to standardise equipment and supplies that would be used in the Brazilian cohort and develop the Preterm SAMBA Standard Operating Procedures (SOPs). One week spent at each university provided the general manager with detailed training in all steps of data and sample collection, processing and storage. Overall, this training allowed transfer of expertise from international partners to the Brazilian team, providing resources for the development of Case-Report Forms (CRF), database and protocols for the Preterm Samba Study. Concern about the temperature control system, institutional adaptation to biobank monitoring (development of maintenance service protocols) and emergency protocols for freezer failure were also considered at this point in time.

Seven different SOPs were designed to standardise several procedures for all related topics, in addition to registering and providing detailed information on such procedures. The following SOPs were created in press and pdf files, with images, photographs, and print screens and provided to all assistants and researchers in all participating centres: (1) Operation Manual: introduction to the database system and details of its functions; (2) Interviewer: information on study design, clinical visits, eligibility criteria, invitation and inclusion procedures, data collection, and definition of all variables; detailed support guidelines on how to administer a 24-h dietary recall (with charts on portion size) and skinfold measurements (with detailed information on technical procedures); (3) sample collection: detailed information about sample collection, biological safety procedures, quality control and ethical aspects; (4) sample processing and storage: detailed information on different steps and quality control regarding the biorepository; (5) division of biobank samples: after sample processing, samples from each woman participating in the Preterm SAMBA study generated serum and plasma aliquots.

Samples were temporarily stored in the local centres, and then shipped to the coordinating centre, which was responsible for dividing the biobank in two—Biobank Brazil and International Biobank. Aliquots from the international biobank were shipped to the international project collaborator for use in previously designed research analyses and also for future discussion by the project steering committee [4]. Careful safety procedures were employed to avoid misclassifying aliquot tracking positions, according to the respective participant and also assure quality control. Such procedures had to be planned and carried out carefully, without any haste. Two serum vacutainer tubes, for instance, were collected from each participant. After processing, serum samples from the second tube were stored in positions immediately after the first tube. Since the biobank was divided in two, alternate aliquots were selected for the Brazilian biobank instead of selecting the first half of the aliquots, for example. Although tricky, this procedure ensured that fibrin or haemolysis of one of the serum vacutainers tube would not compromise the entire Brazilian or International biobank; (6) sample transportation: details on sample packing, parcel configuration, proportion of boxes/aliquots, and dry ice and data logger disposal were described. A certified company in compliance with international rules and recommendations for biological transport in ultra-low temperatures was responsible for national and international sample transport.

### 3.1. Implementing

The Preterm SAMBA biorepository was based on the Institutional Biobank of the Maternity Hospital of the University of Campinas. We believe that institutional support with technical equipment and human resources are crucial to establish a high-quality biobank and allow implementation of the clinical study. A strong commitment of the institution facilitates the following: (1) general and specific arrangements for project implementation and progression: some institutional arrangements are usually required for project implementation such as private rooms dedicated to clinical visits and interviews for the study, changes in patient care workflow, adaptation of routine antenatal care agenda with activities foreseen in the project (sample collection, interviews, etc.), and temporary clinical licence for research assistants (respecting adequate background), allowing their access to medical records at different sites of the institution; (2) identification of possible new assistants throughout the study, to allow for needed reposition: the Brazilian Network experience showed us that despite an occasional replacement of research assistants, this may have a huge impact on study implementation and progress due to possible delays in identifying new suitable collaborators and the need for new training sessions and accreditation. Part-time contract employees from the institution and postgraduate students linked to the project are the most common assistants that collaborate with the project. In our opinion, the main barriers to identify new assistants and the reasons for discontinuation may be related to the perspective that research collaboration may require more strict commitment in terms of workload, quality control, and responsibilities compared to other more profitable activities. Therefore, adequate funding allocated for research assistants and availability of institutional staff to conduct research activities is a priority of great importance; (3) optimal use of resources: the acquisition of new equipment or consumables that will be part of the heritage of the institution should take into consideration existing infrastructure. After the end of the study, the institution will be responsible for the maintenance and supply of consumables.

Some of the equipment and consumables for the biobank were imported. There were few suppliers that provided new solutions in biobank technology in Brazil. Cryovials made of polypropylene, RNase/DNase-free, and pyrogen-free, containing a unique 2-D Data-Matrix Bar Code Insert at the bottom of the vial were used to store serum and plasma aliquots. This technology permits scanning of a whole cryobox with up to 96 cryovials in less than 10 seconds, saving time, and minimizing human errors during the process. In addition, the compact cryobox size results in at least 50% more space in the freezer when compared to ordinary biobank boxes available in Brazil. Depending on the configuration of freezer racks (using low profile racks), space can improve up to 150%. The timeframe to complete all customs clearance procedures for imported equipment and consumables can take months, sometimes years, depending on the item. It is considered a bureaucratic barrier for the Brazilian scientific community. In 2004, an alternative to the conventional import procedure was launched by a joint of Brazilian organisations (the Brazilian official postal service, the Brazilian Ministries of Science, Technology and Innovation and Finance) termed “Science Easy Import system.” Although there are some restrictions on certain items, including value of the commercial invoice, size and weight of the parcel, etc., the system provides a much quicker, less bureaucratic, and more suitable operation. We have translated our experience with the Science Import into a tutorial document to assist and encourage other researchers to use the system [16].

For adequate referral of study subjects, the public health system was strongly involved, raising awareness about the study. It also developed mechanisms aimed at identifying eligible cases, especially within primary healthcare, which was the targeted study population. This strategy was effective, since participating maternities are referral centres for high-risk pregnancies. The Brazilian public health system is decentralised and municipalities provide women with comprehensive, free and universal access to healthcare [17]. Primary healthcare facilities are crucial to the provision of medical care. These units are the basis for the entire healthcare system and a referral for screening. Therefore, antenatal care for low-risk pregnant women should be provided at this level of care. During study implementation, the participating centres arranged local agreements and obtained respective approval of the municipalities to identify eligible women seeking prenatal care at primary healthcare units.

All multicentre studies carried out by the Brazilian Network were launched during the initial meeting for implementation and training with all research assistants, local PIs, research collaborators and funders. Although some training sessions could be organised by webinar meetings, we believe that the interaction and participation of the whole group can transform a seminar into a much more collaborative meeting, with a greater commitment of everyone involved. For the current study, a two-day meeting was held at the University of Campinas to discuss the final version of an electronic CRF within the database and potential local adaptation of any protocol procedure. Furthermore, the study protocol, study equipment, platform for data collection, and a general view of SOPs were also introduced. On-site visits prior to the study were also performed in all participating centres, since sample collection, processing, and storage training sessions required hands-on practice.

A test version of the database, very similar to one that is routinely implemented, was used for training research assistants. In addition, the coordinating centre arranged webinars to discuss more detailed information on the definition of variables and monitored data entered by assistants in the test database during the training period. Only then would the assistants and local PI receive the live database login and password. Passwords were issued following hierarchy, depending on the role of each staff in the study. For instance, a local assistant only had access to data of the correspondent centre, and not to full data.

It is noteworthy that no matter how well-planned a study is, there are possible surprises and challenges while the study is running. In our case, an important fact that could potentially have an impact on the cohort was the Zika virus outbreak that occurred in the country from 2015 to 2016 [18, 19]. As soon as the issue arose, the steering committee decided to include variables that could address the occurrence of the infection, such as history of fever, exanthema, conjunctivitis, and fetal malformations [18, 19]. A few of these variables were already part of routine collection. Nevertheless, the centres were further contacted to pay careful attention to these variables.

### 3.2. Running

**Figure 2:**
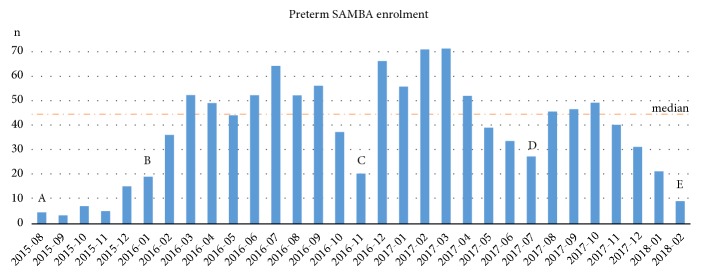
Enrolment rate of the Preterm SAMBA study. Marks A, B, C, D, and E were remarkable set points of the study. The dashed line corresponds to the median – 45.0.

We regard the onset of participant recruitment as the key phase of a prospective study. Breaking through the barrier of initial inertia, reaching the expected enrolment rate, and ensuring that trained procedures are adequately employed are the first challenges to overcome.  Figure 2 demonstrates the enrolment rate of Preterm SAMBA study. Set points, defined as A, B, C, D, and E in the figure, are most important to understand how enrolment progress occurred. We decided to first run the study in only one centre and chose the coordinating centre (A–Figure 2). We were able to monitor the database, recruitment strategies and standard procedures, learn the proper approach to eligible women who might participate in the cohort study with biobank sample collection and the identification and management of potential barriers. This experience enabled more effective on-site training sessions of the remaining participating centres in the following months (months between A and B – Figure 2). As soon as all participating centres started running the project, fortunately it did not take long to reach the expected enrolment rate plateau (B–Figure 2). There were three moments of noticeable decrease in enrolment rates (C, D, and E–Figure 2). Reasons for the decreased inclusion rate of participants in these periods were as follows: C–temporary absence of one of the two research assistants in one centre and a hospital staff strike in another centre; D–change of research assistants in two participating centres and a technical issue with the freezer in one of these centres (new equipment had to be bought); E–a decrease in enrolment rate was evident in the last few months of the study, most likely due to the long period of data collection (over two years) and distance from sites where visits or group meetings took place. We believe that constant monitoring and surveillance, not only remote feedback, are important to maintain the inclusion rate of cases in such cohorts. Furthermore, ongoing shifts in initially defined strategies to identify and recruit eligible women should also be considered.

Television, radio, and newspaper media and a website containing general information about the study, profile of eligible women, and contact of all participating centres were the main strategies employed to draw attention to the project and reach a local and regional audience.

A general project manager was designated to perform continuous monitoring of quality control parameters (data and sample collection; sample processing and storage), monitoring of freezer temperature records, inclusion, and loss to follow-up rates and inconsistent dataset. Two or three monitoring visits were made to each centre during the study to provide feedback on study procedures, difficulties, and barriers to run the study and repeat some training sessions, according to the performance of the centre and the evaluation of the monitor. A standard monitoring report was written after each visit and shared with all local researchers, assistants and also the coordinating centre, to register established commitments and decision making on how to overcome difficulties encountered. Main parameters reported were the number of included participants per month, loss to follow-up, missing data, data inconsistencies, SPREC (Standard PREanalytical Code) pre-analytical quality parameters for biological samples [20], number of aliquots generated per tube, occurrence of fibrin, time between collection and storage, strategies for identifying eligible women, and others. In addition to on-site monitoring visits, several web-conference meetings were performed to discuss the details of study progress and data inconsistencies, enabling continuous monitoring and a learning atmosphere which facilitated the identification and correction of human errors and unproductive work processes.

## 4. Discussion

### 4.1. Monitoring

An open, two-way communication between research assistants of all participating centres and the general study monitor was of the utmost importance for the establishment of an effective organisation and coordination of the study. We did not expect the lack of mistakes during procedures or human errors discordant from standard protocols. Nevertheless, it is not only important to prevent these errors, but also to recognise, report, and adequately record the issues. It is helpful to accept errors in a nonpunitive atmosphere to enhance the confidence of collaborators. In terms of communication, cell-phone group chats were the quickest and most objective method employed to assure clear and quick-response communication. Following ethical principles, no data of participants were ever shared through message chats.

The database system was developed in the MedSciNet® platform, a resourceful and very helpful online system for study management. It securely links clinical data to biobank data, providing hierarchical access, a biosample tracking system, and multiple functions for data monitoring. At the end of the study, the system provides a case-control function through which the user can design clinical experiments, selecting women whose sample positions will be provided.

According to study protocol, all women who were invited to participate in the study and refused to undergo complete or partial sample collection were properly registered. Contrary to what we had anticipated, eligible women rarely declined or reported feeling uncomfortable with blood and/or hair collection.

Regardless of research team support or antenatal care provided by the participating institution, a few women preferred to maintain their antenatal care visits in primary healthcare facilities or continue with their own private doctors. Reasons were either emotional because of a good relationship previously established with healthcare providers or more practical due to a closer distance from the primary care unit. This caused a little but noticeable impact on rates of inclusion and loss to follow-up, owing to delays in attending scheduled visits for sample collection. In addition, the staff found it a much more difficult and time-consuming task to complete information on women delivering outside the participating facility, and required specific local strategies. We noticed that centres which provided ANC visits had better performance in rates of inclusion and loss to follow-up.

Public health facilities where eligible women were identified and invited to participate had different reactions to the study. Some units were helpful and highly committed to the task, facilitating study processes and establishing clear boundaries between research and healthcare activities. According to strategies designed to ascertain adequate follow-up of included cases, the centres were encouraged to provide ANC support. Although complete healthcare was not the responsibility of the study, as previously mentioned, the proportion of women who agreed to participate and adhere to the study was traditionally higher in centres that offer a personalised ANC assistance.

Regardless of the ANC provided, research assistants were trained to adequately report several maternal and perinatal outcomes. Standard outcomes—PE (pre-eclampsia), GDM (gestational diabetes mellitus), PTB (preterm birth), and FGR (fetal growth restriction)—were consistently checked against previously standardised diagnostic criteria. Retrospective data review to confirm or rule out an outcome can be a challenge, since it may underestimate or overestimate the incidence of the outcome. Taking into account GDM, PTB, FGR, and PE, we believe that PE is the most challenging outcome to review and double-check. Registration of high blood pressure levels and urine protein values during study visits might not suffice to assure its occurrence [21]. Participating institutions have to follow an adequate standard obstetric protocol to ensure that late pregnancy and delivery/postpartum data will be appropriate to confirm PE cases. Nonstandardised recording may introduce bias and generate uncertainty with compromised data. In summary, the concept “do it right the first time” is legitimate and strongly recommended, as well as the application of international standard definitions during the study protocol. For these data, it is also important to know the clinical protocol of each institution and reinforce the importance of its use.

The provision of national and international biosample transport was planned in two steps. First, for national sample arrangement, each participating centre sent biosamples to the coordinating centre when 60% of the sample size was attained. Then, a second transport was arranged, containing the remaining samples. After shipment from participating centres arrived, the position of each cryobox containing biosamples was updated in the database system, according to the freezer of the coordinating centre. Transportation was provided by an experienced company in compliance with IATA (International Air Transport Association) and international ethical regulations. Samples were maintained in dry ice and monitored by a temperature data logger in each parcel. The preparation for international shipping entailed further splitting of the samples into International Biobank and Biobank Brazil, as previously detailed. The first international transport contained 80% of the International Biobank samples. Consequently, the second contained 20% of samples.

The coordinating centre held a final meeting with all local investigators, research assistants and collaborators to discuss the plan for analysis of primary and secondary outcomes. The participation of all collaborators from all participating centres was truly important not only to put into practice the opportunity to collaborate among all researchers and assistants involved in the study but also extend the exploration of secondary analysis by using a comprehensive dataset. Investigators that built their careers in different areas of maternal and perinatal health research were able to share their experience and suggest innovative approaches to examine some specific complications that occur during pregnancy. Moreover, participating centres of the Brazilian Network contributed to the discussion about the implementation of the next big study, termed the Maternal Actigraphy Exploratory Study–I (MAES-I). The future study is an extension of the Brazilian multicentre cohort study. It will investigate physical activity and sleep patterns throughout pregnancy, in association with maternal complications using actigraphy data (study protocol to be published). Human and technological resources allocated for the Brazilian multicentre cohort using biobank resources may facilitate the implementation of new studies and application of innovative approaches including wearable technologies, such as the actigraphy device to monitor women during pregnancy.

## 5. Conclusions

Planning, implementing and running a multicentre study with standardised clinical data and biosample collection is challenging, although possible in low-income and middle-income settings. Proper funding, with a detailed developed proposal and well-established network, is pivotal to the development of such an initiative. Enhancement of capacity and training in settings most commonly affected by the burden of disease in maternal and perinatal health will strengthen findings and provide a venue for future research.

The Brazilian Network, created almost a decade ago, with prior conduction of successful studies was fundamental to support the current initiative. Furthermore, funders willing to collaborate throughout the study create an opportunity for more than study results and reports. There is room for more effective learning to disseminate findings, raise awareness about research impact, and add new perspectives and collaborations.

## Data Availability

The current manuscript describes several topics related to methodological aspect and the implementation of a multicentre cohort study and does not involve data to be available.

## Abbreviations

ANC: Antenatal care
BMGF: Bill and Melinda Gates Foundation
BMI: Body mass index
BNSRPH: Brazilian Network for Studies on Reproductive and Perinatal Health
CEP: Local Institutional Review Board
CNPq: Brazilian National Research Council
CONEP: National Committee for Ethics in Research
CRF: Case-report forms
FGR: Fetal growth restriction
GDM: Gestational diabetes mellitus
IATA: International Air Transport Association
IMPROvED: IMproved Pregnancy Outcomes by Early Detection
IRB: Institutional Review Board
PE: Preeclampsia
PI: Principal investigator
PTB: Preterm birth
Preterm SAMBA: Preterm Screening and Metabolomics in Brazil and Auckland
SCOPE: Screening for Pregnancy Endpoints study
SOP: Standard Operating Procedures
SPREC: Standard PREanalytical Code
UNICAMP: University of Campinas
Wk: Week.
